# Special Collection: Protocols in Medical and Veterinary Entomology

**DOI:** 10.1093/jisesa/ieaa122

**Published:** 2020-11-02

**Authors:** Erika T Machtinger, Karen C Poh

**Affiliations:** Department of Entomology, Pennsylvania State University, University Park, PA

Arthropods of medical and veterinary importance live alongside people, our livestock and pets, and wildlife, creating a pathway for zoonotic pathogens to jump between reservoir hosts and accidental hosts. These pests and parasites at a minimum are the sources of general annoyance to people and animals. Some serve as vectors which inflict further damage by transmitting pathogens that cause disease and negative changes in body condition or physiology to hosts. In addition to biological impacts, pests of livestock can cause billions of dollars in economic damage (Kunz et al. 1991). Furthermore, vector-borne diseases are on the rise, with the CDC reporting in 2018 a tripling of disease cases transmitted by mosquitoes, ticks, and fleas in the past 13 yr (Rosenberg et al. 2018).

Studies of these organisms and how to reduce their impacts on animal and human welfare began ‘in the field’, in the natural or synanthropic habitat occupied by the pest. However, the field techniques to trap, study, or manipulate pests of medical and veterinary importance have not been described as thoroughly as molecular and ‘-omics’ methods. Many field techniques are not fully detailed in manuscripts but are instead housed with the developer and not shared publicly. Some of these techniques and procedures may be lost over time, as a result of developer attrition (e.g., retirement, career change, death). Thus, we present herein a collection that provides protocols, procedures, and techniques for many important field components of pest research and includes studies of common pests of humans and animals, as well as some lesser-studied organisms.

The use of recommended field techniques and protocols provides consistency across studies and allows for comparisons of data even across different geographies. A collection of such protocols and techniques serves as a reference point for students and other scientists across many institutions (e.g., government, academia, or industry) who are beginning research on pests of medical and veterinary importance. Many of the papers in this collection also provide considerations and potential pitfalls that researchers should be aware of before, during, and after data collection, which will expedite the research study overall. The goal is to preserve and catalog protocols to be shared with generations of future medical and veterinary entomologists.

The papers in the special collection include:

- Oral and Topical Insecticide Response Bioassays and Associated Statistical Analyses Used Commonly in Veterinary and Medical Entomology (Burgess et al. 2020)
- Sampling Efficacy and Survival Rates of *Labarrus pseudolividus* (Coleoptera: Scarabaeidae) and *Onthophagus taurus* (Coleoptera: Scarabaeidae) Using Flotation and Sieve-Separation Methodology (Fowler et al. 2020)
- Improved Sentinel Method for Surveillance and Collection of Filth Fly Parasitoids (Geden et al. 2020)
- Monitoring House Fly Activity on Animal Facilities (Gerry 2020)
- Laboratory Methods for Rearing Horn Flies (Diptera: Muscidae) (Holderman et al. 2020)
- *Beauveria bassiana* Culturing and Harvesting for Bioassays With House Flies (Johnson et al. 2020)
- Practical Guide to Trapping *Peromyscus leucopus* (Rodentia: Cricetidae) and *Peromyscus maniculatus* for Vector and Vector-Borne Pathogen Surveillance and Ecology (Machtinger and Williams 2020)
- Sampling Considerations for Adult and Immature Culicoides (Diptera: Ceratopogonidae) (McDermott and Lysyk, 2020)
- Collecting and Monitoring for Northern Fowl Mite (Acari: Macronyssidae) and Poultry Red Mite (Acari: Dermanyssidae) in Poultry Systems (Murillo and Mullens 2020)
- Collection and Rearing of Container Mosquitoes and a 24-h Addition to the CDC Bottle Bioassay (Parker 2020)
- Collecting Deer Keds (Diptera: Hippoboscidae: *Lipoptena* Nitzsch, 1818 and *Neolipoptena* Bequaert, 1942) and Ticks (Acari: Ixodidae) From Hunter-Harvested Deer and Other Cervids (Poh et al. 2020)
- Trapping White-Tailed Deer (Artiodactyla: Cervidae) in Suburbia for Study of Tick–Host Interaction (Roden-Reynolds et al. 2020)
- A Beginner’s Guide to Collecting Questing Hard Ticks (Acari: Ixodidae): A Standardized Tick Dragging Protocol (Salomon et al. 2020).
- A Technique for Dissecting the Salivary Glands From the Abdomens of Deer Keds (Diptera: Hippoboscidae: *Lipoptena* Nitzsch, 1818 and *Neolipoptena* Bequaert, 1942) (Skvarla et al. 2020)
- General Considerations for On-Animal Ectoparasiticidal Product Evaluations (Smythe and Sanchez-Sandoval 2020)
- Using Visual and Digital Imagery to Quantify Horn Fly (Diptera: Muscidae) Densities (Smythe et al. 2020)
- Methods for Surveying Stable Fly Populations (Taylor et al. 2020)
- A Tissue Digestion Protocol for Measuring *Sarcoptes scabiei* Density in Skin Biopsies (Tiffin et al. 2020)
- A Simple, Inexpensive Method for Mark-Recapture of Ixodid Ticks (White et al. 2020)
